# Collaborative networking among agricultural production cooperatives in Iran

**DOI:** 10.1016/j.heliyon.2022.e11846

**Published:** 2022-11-26

**Authors:** Mohammad Alimohammad, Seyed Jamal Farajallah Hosseini, Seyed Mehdi Mirdamadi, Sahar Dehyouri

**Affiliations:** a Department of Agricultural Economics, Extension and Education, Science and Research Branch, Islamic Azad University, Tehran, Iran; b Agricultural Extension and Education, Islamshahr Branch, Islamic Azad University, Islamshahr, Iran

**Keywords:** Network, Agricultural production cooperatives, Mechanism, Iran

## Abstract

This research seeks to determine mechanisms in developing collaborative networking among agricultural production cooperatives in Iran. The research design was survey method and a network consisting of agricultural cooperatives has been defined as the core of the network. The statistical population of the study included managers of cooperatives which were administrated by the “Ministry of Cooperatives, Labour and Social Welfare” and the “Rural Cooperative Organization” of the Alborz Province (N = 540). From this target population, 176 managers of cooperatives were selected by multi-stage cluster sampling. A questionnaire was developed to determine the technical, economical, educational, legal, social, and policy making mechanisms that presented the collaborative networking model. The validity of the questionnaire was confirmed by experts in the field of Agricultural cooperative. To calculate the reliability coefficient of the questionnaire, Cronbach’s alpha coefficient, which is used for multi-scale questions, was used. The mean of Cronbach’s alpha coefficients in this study was 0.803. The results showed that policy making with a coefficient of 0.539, economic with a coefficient of 0.499, legal with a coefficient of 0.208, and educational mechanisms with a coefficient of 0.130 had the most impact, while technical and social mechanisms had no role in developing the collaborative networking.

## 1 Introduction

Organizations interact with the environment and with other organizations, and these interactions constitute an important way of learning and evolution. To overcome the problems that they face during their existence, organizations must certainly adopt survival strategies, both individually and in group (Lopes et al., 2020).

Networking has several definitions that relate to issues such as formal and informal relationships within and outside the organization between individuals and organizations and display behaviours that play a role in establishing these relationships.

Both inter- and intraorganizational networks draw the attention of researchers from various disciplines who view them as the fabric of the socioeconomic world (Sliwa, 2022).

Organizational networks are often seen as a new way to get things done. In many instances outcomes cannot be realized by an individual organization because the task is complex (in contrast to simple or complicated tasks) and/or when tailor-made services are demanded (Kenis and Raab, 2020).

Gould and Penley (1984) believe that networking is the process of establishing a system that enables internal and external communication between individuals and organizations. Formal networks define relationships between groups based on their tasks to achieve organizational goals (Ibarra, 1993).

Jane Wei-Skillerna professor of business management at Harvard University, examines the role of networking in NGOs, saying the networking deals with a set of actors and the relationships between them (HBS, 2005).

Networking and Linkages focus on very good communications and partnerships with others to serve people with mutual interest. It is another important part of management to strengthen its organization to develop and manage its communications to networking with other organizations or having joint projects with others in assisting vulnerable community (Nanthagopan, 2011).

Gaonkar and Mele (2019) by reviewing literature about networking examined the impact of networking on the performance of organizations, development of innovation, access to resources and manage the competition.

Various studies show the effects of networking on knowledge management and learning in the organization. Turner and Pennington (2015) in a study examine knowledge sharing and organizational learning as a mean to drive entrepreneurship and innovation in organizational networks. The results show that knowledge sharing, and organizational learning are associated with the motivation, opportunity, and ability to act within the corporate entrepreneurial context. Emmerik et al. (2006) in a study pointed out about importance of networking that provide its members with added information, mentoring, and political support.

Collaboration is considered as one of the principles of success in today’s business. The importance of collaboration stems from the fact that it has led to the exchange of information, customer - focus and competition (Beyerlein et al., 2003). Dean (2010) in a working paper about intra organizational collaboration, citing Msanjila and Afsarmanesh (2008), defines collaboration the exchange of risks, resources, responsibilities, and information that requires mutual participation and trust in achieving common goals.

Collaboration has positive effects on stakeholder participation in implementing organizational strategies. In its strategic document, the Council on Linkages Between Academia and Public Health Practice emphasizes the importance of collaboration and emphasizes that this collaboration can pave the way for the involvement of all stakeholders (PHF, 2016).

Collaboration would also strengthen the human resources to have access to capacities and abilities to strengthen their complementary role and encourage them share responsibilities (Syed Kechik, n.d.).

Collaboration between multiple agencies isn’t always easy, but it’s always worth it. Along the way there may be a few bumps; overlapping capabilities, different financial goals, and even egos can make collaboration and integration a full-time job on its own. But, with the right goals, approach, clear accountabilities, and willing partners, it can be done – and done well (Young, 2017).

Participation of rural population in agricultural activities and the proper use and fair distribution of resources and opportunities is an effective strategy of modern and developed societies to empower rural communities (Karami and Rezaei Moghadam, 2005). Organized and voluntary participation of villagers is conducted through different type of organizations such as cooperative. Such organisations represent the interests of different segments of the population. This attitude leads to the introduction of cooperative producers as one of the most important manifestations of organized agricultural involvement in economic and social affairs.

Participation is the basis of cooperative networks; thus, it could be defined as the process of voluntary, conscious, and intentional decision-making individually or collectively to empower oneself to meet specific needs and goals in a particular situation, which can be performed spontaneously or planned.

Reed and Hickey (2016) citing Bernard and Spielman (2009), and Fischer and Qaim (2012) refer to the importance of cooperative to facilitate information exchange, improve collaboration, innovation, and market access for smallholder farmers.

Many studies have been carried out regarding the role of cooperative producers on empowerment of member farmers and agricultural development. Some of these studies have shown the positive effects and consequences of agricultural cooperative producers and some others have addressed the challenges and problems faced by the cooperatives.

In general, rural population can improve efficiency of their activities and enhance their economic situation by performing professional activities based on participation and cooperation (Nasiri, 2010).

Furthermore, organizing farmers in the form of associations and cooperatives can provide the ground for their empowerment and enhance their skills and abilities in implementing agricultural policies (Kassie et al., 2013).

Furthermore, Swanson (2008) in his research suggested that the organisation of different communities of farmers and the formation of local unions will secure food accessibility of local communities and lead to the development of them.

However, the structure of cooperatives is becoming more product based and tend to be more farmer owned. To be more competitive and international, many cooperatives are introducing managerial entrepreneurship (Karakas, 2019). To be more effective, competitive, and resilient, cooperatives should form networks among themselves based on the type of services and products that are delivered.

Establishing networks in the agricultural sector is one of the important factors in agricultural development and significantly reduces the vulnerability of the cooperatives in the face of crises (Karimi Goghari et al., 2017).

The results of the study about performance of cooperatives society in Latvia revealed that the main threat to the development of cooperative societies was the political factor – possible sudden and significant changes in the national agricultural policy, which might be affected by the turnover of policy makers and the priorities set for the industry, as well as the economic situation in the country (Mistris et al., 2020).

Governments are expected to provide a supportive policy, legal and institutional framework, provide support to cooperative societies based on activities, provide oversight on terms equivalent to other forms of enterprise and social organization, adopt measures to improve access to finance for disadvantaged groups, and topically, to promote the formalization of the informal economy (Dogarawa, 2005).

In Iran, along with many other developing countries, the formation of non-governmental organizations has grown significantly in the last two decades. Non-governmental organizations and institutions have different identities, affiliated legal and regulatory authorities. These different identities include social identity (related to the laws of the Ministry of Interior and the laws governing parties and social institutions), scientific identity, commercial identity (related to the Ministry of Industry, Mine and Trade), economic identity (cooperatives under the Ministry of Cooperatives and the Organization of Rural Cooperatives) as well as public and private companies and institutions and trade unions under Ministry of Agriculture.

Currently, there are more than 1800 agricultural cooperatives registered in the agricultural sector alone, most of which have been formed in less than two decades (Statistical Yearbook of Ministry of Cooperative, 2018). To further collaborate among these cooperatives, a network of 59 unions have been organized based on different agricultural activities.

To strengthen and coordinate among these cooperatives, it will be necessary to develop a systematic network coupled with the support of the government so that producers will be encouraged to participate in this network and simultaneously government services will be provided within the network. This situation necessitates the creation of a comprehensive network or structure to cover multiple different units. Accordingly, it is necessary for all non-governmental organizations or at least organizations affiliated to the agricultural business to be connected to each other as a single network so that they can have maximum efficiency through their collaboration.

Lin and Lin (2016) investigated the relationship between networks and the performance of small and medium enterprises (SMEs) in Taiwan and indicated that the relationship has been positive in different levels of companies.

In another study by Husain et al. (2016) showed that organizational networks lead to competitiveness through organizational learning and innovation. Mitrega et al. (2012) also examined the networking capabilities and their consequences and subsequently introduced networking as a complementary pillar of the network management model.

A review of the experiences of successful companies shows that networking within the organization and outside the organization has influenced the collective learning and innovative performance of these companies Garousi Mokhtarzadeh et al. (2020).

In examining the relationships between networks inside and outside the organization, Birkinshaw (2000) points out that the prerequisite for the success and effectiveness of these networks is related to sufficient skills to coordinate between these networks. In addition, the ability of human resources in an organization in networking plays an important role in facilitating this coordination.

Mazarrol et al. (2013) in a study about role of cooperative as a strategic network based on the case studies from Australian and French cooperatives found out that sustainability of these cooperatives has been dependent upon network management and adaptability along with some other factors such as trust and loyalty of members.

Develtere et al. (2008) while studying the role of cooperative networks in rural areas, found out that cooperatives experience provides the ground to delegate agricultural affairs to a network of cooperatives.

In this regard, Guzman et al. (2017) to analyse the social network approach and to understand organizational capacities and interactions in promoting organic agriculture indicated that inter-institutional communication models increase the institution’s capacity, facilitates their access to information and resources, creates inter-institutional cooperation and improving their ability to achieve their goals.

Designing a network of agricultural cooperatives, while increasing efficiency, reducing transaction costs, and facilitating direct access of customers to producers, maximizing retailer farmers and villagers' share of production’s added value, encouraging the producers, empowering farmers by increasing their share and ultimately reducing defective and unfruitful cycles of brokers.

Formation of networks is affected by many factors and interaction between these different factors eventually lead to more efficient and effective networks. In this regard, examining the factors that form the networks would improve existing relationship between them and provide the ground for more technical, social, and economic cooperation among organization (Gao and Yu, 2022).

Improving economic growth with a focus on increasing cooperation among co-operators requires an impact evaluation of both members and non-members for a respective sector (Sambuo, 2022).

The main purpose of this study is to determine mechanisms affecting the design of a model for the collaborative networking among agricultural cooperatives in Alborz Province.

In this model, all the mechanisms involved in the network are identified and the effectiveness of each is evaluated and measured. Finally, the influential mechanisms are introduced and insignificant one in the measurement model will be eliminated. Furthermore economic, educational, extension, research, social, policymaking, legal and technical mechanisms are examined along with the main objective.

We discuss the potential mechanisms that could have contributed to the development of these networks. Such potential mechanisms are economic, social, policy, educational, technical, and legal. With respect to the lack of collaborative networking among agricultural cooperatives, this survey study aims at answering this question: what are determining mechanisms in development of collaborative networking among agricultural cooperatives?

## 2 Coverage for this study

**Figure 1:**
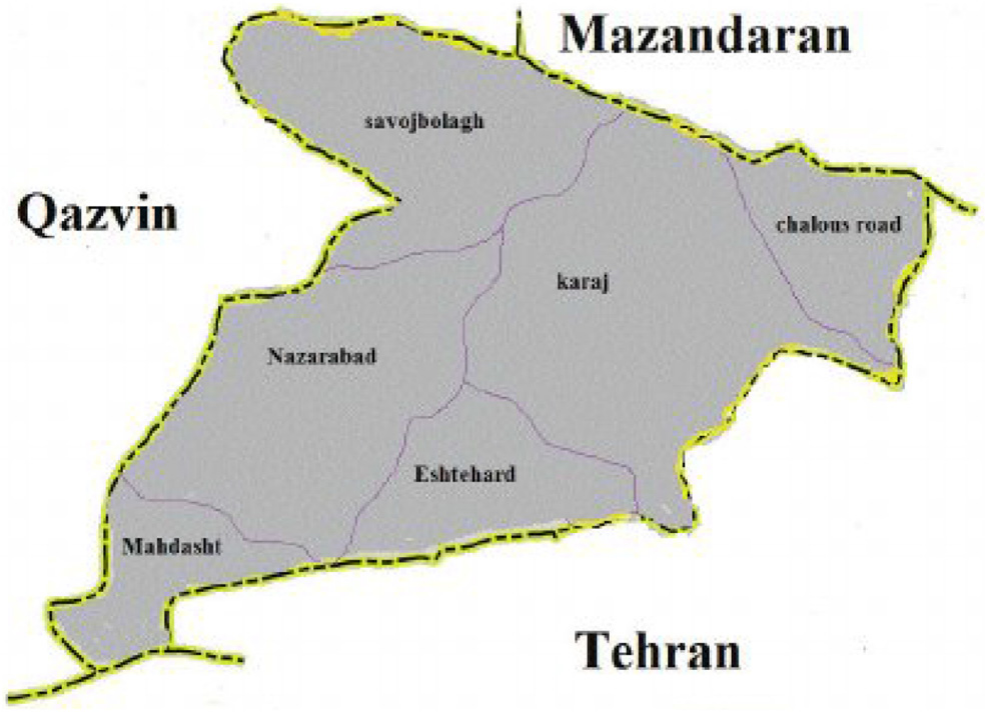
Map of Alborz Province.

Alborz Province was formed by division of Tehran Province into two provinces, after the Parliamentary approval on June 23, 2010, and was introduced as 31st province of Iran. Situated northwest of Tehran, the Province of Alborz has 6 counties, Karaj, Savojbolagh, Taleqan, Eshtehard, Fardis and Nazarabad. According to the National Census, in 2016 population of Alborz was 2,712,400 million out of which 90,5% lived in urban areas. Figure 1 illustrates the map of Alborz province.

## 3 Conceptual framework

**Figure 2:**
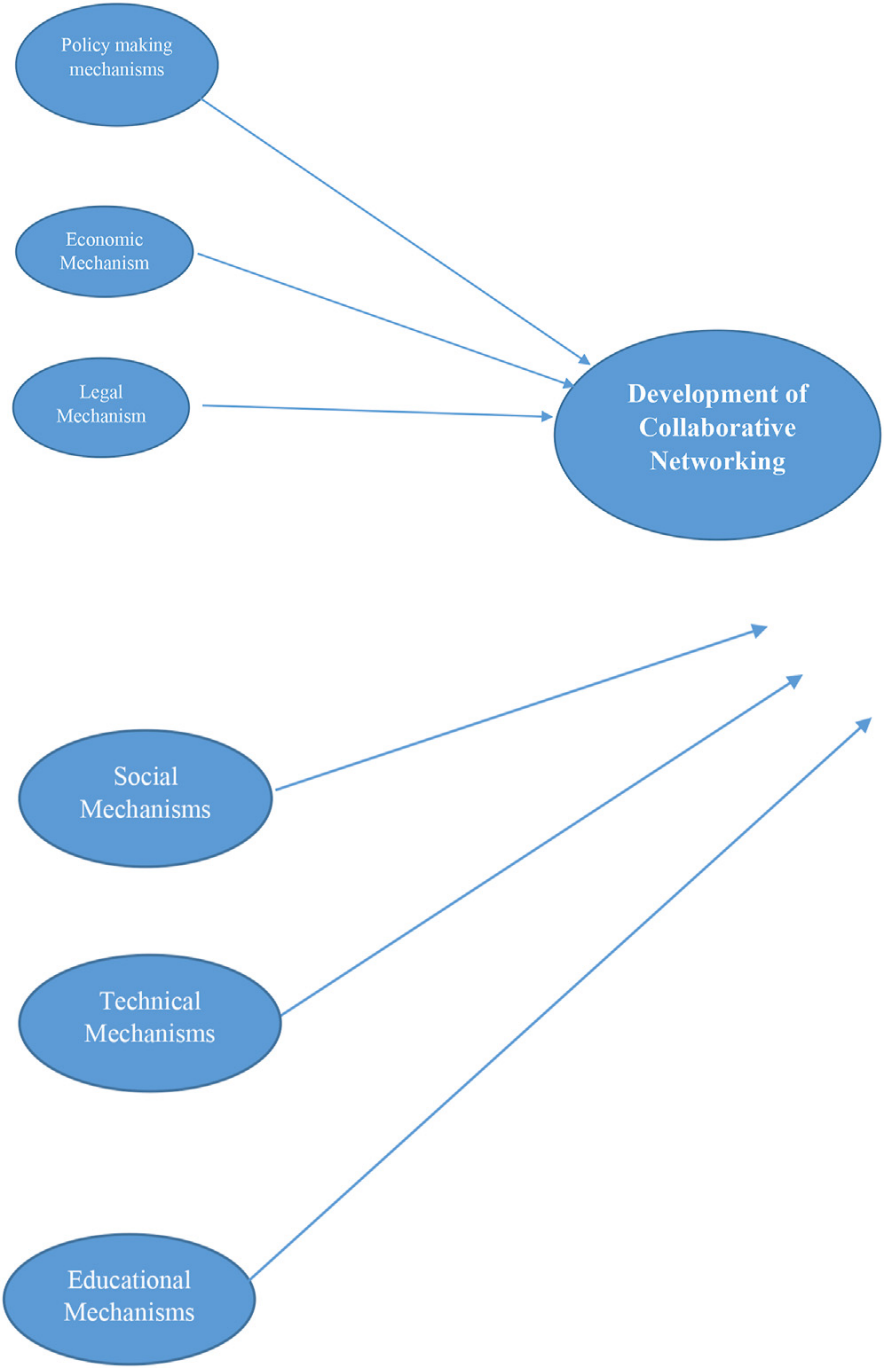
Conceptual framework.

The dependent variable of this study is the development of collaborative networking which consists of network-based behavior, market-oriented behavior, and relationship-based behavior. The mechanisms that contribute to the development of collaborative networking would in six following mechanisms: policy making, economic, legal, social, technical, and educational mechanisms. Moreover, Figure 2 illustrates how these attributes would be designed as mechanisms to the development of collaborative networking among cooperatives.

## 4 Methodology of the research

This study applies a quantitative case study approach, and a survey method is used to collect the data. The study analysed quantitative data that focused on mechanisms that contribute to development of collaborative networking among agricultural cooperatives by collecting field data.

The statistical population of the research consists of managers of agricultural cooperative under the auspices of the Ministry of Cooperative, Labour, and Social Welfare and the Organisation of Rural Cooperatives (a total of 540 cooperatives performing in various fields of production, services, and related agricultural industries) located in Alborz province. Multi-stage cluster sampling method is used to determine the size of the sample. The Cochran formula is used to determine the sample size that essential sample carried up by simple random or unsystematic sampling method. In the formula of the Cochran, p and q are the ratio of success and failure that are considered 0.5. Considering the sampling error rate of 5%, the sample size has been estimated as 176 employing the following formula (Table 1).

**Table 1:** Target population.

Orientation	Number of totals	number of samples
Agricultural production	260	85
Distribution and export	110	35
Alternant industries	106	35
Services and office	64	21
Total	540	176

The formula of Cochran is written as Eq. (1).

(1) $$n=\frac{Nz^{2}pq}{Nd^{2}+z^{2}pq}$$

The value of $Z_{a/2}$ at error level of 0.5 is equal with 1.96.
The error value of d is considered as 0.05.
The N - value defines the volume of the community.

Based on literature review, following hypotheses was presented:

Economic mechanisms contribute to the development of collaborative networking.

$$H_{o}:R=0,H_{1}:R\neq 0$$

Social mechanisms contribute to the development of collaborative networking.

$$H_{o}:R=0,H_{1}:R\neq 0$$

Technical mechanisms contribute to the development of collaborative networking.

$$H_{o}:R=0,H_{1}:R\neq 0$$

Legal mechanisms contribute to the development of collaborative networking

$$H_{o}:R=0,H_{1}:R\neq 0$$

Policy making mechanisms contribute to the development of collaborative networking

$$H_{o}:R=0,H_{1}:R\neq 0$$

Educational mechanisms contribute to the development of collaborative networking

$$H_{o}:R=0,H_{1}:R\neq 0$$

A questionnaire consisting of two parts was designed for collecting data. The first part was about the descriptive data about respondents and second part consisted of questions regarding mechanisms that contribute to development of collaborative networking. Semi-structured interview was used to gather in-depth data from managers of agricultural cooperative.

Exogenous latent variables of this research have been measured using technical, economic, legal, social, educational, and policy-making mechanisms while the endogenous latent variables of our structural equation have been evaluated using following indicators production management, inputs, agricultural products, the role of government agencies and financial institutions, law and regulations of producers, distributors and exporters, the role of non-governmental organizations, cultural, educational, promotional and research factors, economic policies and development.

In the analytical section, structural equation modeling (SEM) was used to determine the relationship, role, effect and comparison of mean differences. The calculations were performed using Amos software. Structural equation modeling (SEM) is a form of causal modeling that includes a diverse set of mathematical models, computer algorithms, and statistical methods that fit networks of constructs to data. SEM includes confirmatory factor analysis, confirmatory composite analysis, path analysis, partial least squares path modeling, and latent growth modeling. SEM is used to show the causal relationships between variables. The relationships shown in SEM represent the hypotheses of the researchers. Typically, these relationships cannot be statistically tested for directionality. SEM is mostly used for research that is designed to confirm a research study design rather than to explore or explain a phenomenon. SEM produces data in a visual display – and this is part of its appeal. When using SEM, the researcher gets a tidy visual display that is easy to interpret, even if the statistics behind the data are quite complex.

The second approach is Partial Least Squares (PLS), which focuses on the analysis of variance and can be carried out using PLS-Graph, VisualPLS, SmartPLS, and WarpPLS. In this study the SmartPLS software was used to analyse the data. Kolmogorov-Smirnov test was also applied to test the normality of the data.

Wong (2013) in a technical paper indicated that there are several distinct approaches to SEM: The first approach is the widely applied Covariance-based SEM (CB-SEM)6, using software packages such as AMOS, EQS, LISREL and MPlus.

To test the reliability of the measurement model factor loadings, combined reliability (CR), convergent validity (AVE), divergent validity, R^2^ criterion, Q^2^ criterion, Redundancy criterion and GOF criterion were applied.

Significance coefficients of Z (t-values) were also used to determine the nature of the relationships between the constructs and to see whether the research hypotheses are confirmed or rejected.

## 5 Results

A brief descriptive statistical analysis of sample showed that the average age of the managers was 45 and more than 80 percent were male. It was also observed that 68% of the respondents had at least a bachelor’s degree and the average of work experience in cooperative was 18 years.

In the structural model analysis, considering the significance level being smaller than 0.05, which was measured by Kolmogorov-Smirnov test, it is indicated that the variables have not been normal. Regarding size of the sample, the researcher concluded to use the structural equation modelling method (PLS) to analyse the data.

**Figure 3:**
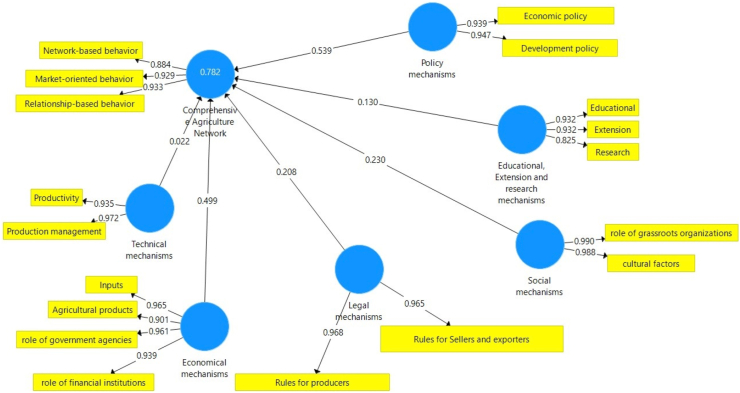
Factor loading coefficients.

Figure 3 shows diagram of factor loading coefficients as the first output of the PLS structural model. The results show that relationship-based behaviour among three variables related to networks among cooperatives with 0.933 factor loading had the highest impact.

To examine the reliability and validity of the model, these criteria were evaluated:

1. **Factor loadings analysis:** Factor loadings are measured by calculating the correlation value of the indicators of a construct with the construct itself. If this value is equal to or greater than 0.4, the model will be reliable. Based on the output of software, this value is greater 0.4, so the reliability of the model is approved.
2. **Composite reliability:** this criterion measures the reliability of constructs not in an absolute way and the correlation of constructs with each other shows the value is greater than 0.7 (Table2).

**Table 2:** Composite reliability and convergent validity values.

Mechanisms	Combined reliability CR	Convergent validity AVE
Educational, Extension and research mechanisms	0/926	0/806
Social mechanisms	0/989	0/978
Legal mechanisms	0/966	0/934
Technical mechanisms	0/953	0/909
Policy mechanisms	0/941	0/889
Economical mechanisms	0/969	0/887
Collaborative Networking	0/941	0/838
3. **Convergence Validity (AVE**): Convergence Validity (Average Variance Extraction) indicates the degree of correlation of a construct with its characteristics and the higher the correlation, the greater the fit of the model. Convergent validity is acceptable if its values are greater than 0.5 (Table 2). The CR value for these mechanisms was more than 0.70 and AVE value was more than 0.5 indicating the reliability and validity of model.
4. **Divergent Validity:** Divergent validity is a criterion to check the fit of the measurement model in PLS. In this study, the Fornell-Larcker method was applied to measure the relationship between a construct and its characteristics in comparison with the relationship between a construct with other constructs. Divergent validity is examined by a matrix and is acceptable if the values on the main diagonal are greater than the values below them (Table 3).

**Table 3:** Divergent validity matrix by Fornell and Larker methods.

Collaborative Networking	Economic mechanisms	Policy mechanisms	Technical mechanisms	Legal mechanisms	Social mechanisms	Educational, Extension and research mechanisms	
						0/898	Educational, Extension and research mechanisms
					**0/989**	0/806	Social mechanisms
				**0/966**	0/841	0/750	Legal mechanisms
			**0/954**	0/630	0/761	0/739	Technical mechanisms
		**0/943**	0/605	0/670	0/605	0/607	Policy mechanisms
	**0/942**	0/623	0/771	0/822	0/944	0/823	Economic mechanisms
**0/916**	0/790	0/784	0/680	0/672	0/764	0/653	Collaborative Networking
5. **R**^**2**^**criterion:** this criterion connects the measurement and structural models in the structural equations modelling and indicates the effect that an exogenous variable has on an endogenous variable. The value of R^2^ is only calculated for endogenous (dependent) variable of the model and for exogenous constructs, this value is zero. Three values of 0.19, 0.33, 0.67 are considered as weak, medium, and strong impacts, respectively.
6. **Q**^**2**^**criterion:** This criterion determines the goodness of predication of model and the values of 0.02, 0.15 and 0.35 indicate weak, medium, and strong goodness of predication of the variables, respectively. This value is only Calculated for endogenous variables of the model.
7. **Redundancy criterion:** This criterion indicates the amount of variability of the indicators of an endogenous construct which is influenced by one or more exogenous constructs and the higher the average of Red, the more appropriate the fit of the structural model of the study.

Red = Communality × R^2^

8. **GOF criterion:** this criterion could be examined for both measurement and structural models. Values of 0.01, 0.20 and 0.36 will be described as weak, medium, and strong, respectively.
9. **t-values:** This is the most basic criterion to measure the relationship between variables in the model. If the value is greater than 1.96 the accuracy of the relationship between the constructs is confirmed and thus the research hypothesis is valid at a 95% confidence level. Of course, it should be noted that this criterion indicates the correctness of the relationships while the intensity of the relationship between the constructs cannot be measured through it.

**Figure 4:**
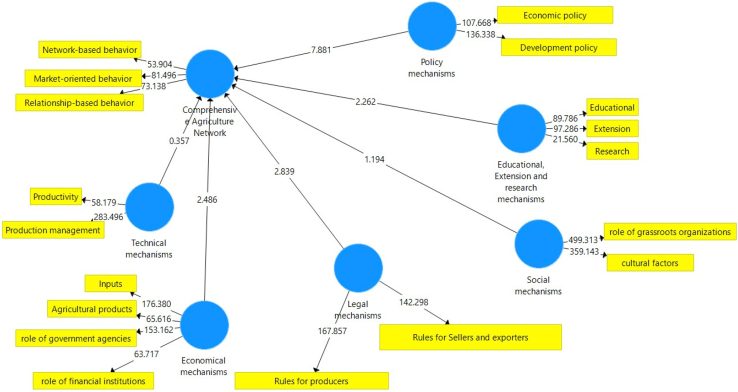
The diagram of significant coefficients of z.

Figure 4 shows the structural model with standardized coefficient and policy making mechanism with the t value of 7.881 has the highest impact and technical mechanism with 0.357 has the lowest impact on the development of collaborative networking among cooperatives.

Based on the results of the study, in examining policy-making factor and, two substantial indicators of development policies and economic policies, the development policies in comparison with economic policies was a stronger determining factor in the development of collaborative networking in cooperatives.

To analyse the economic mechanisms, four indicators of inputs, role of government agencies, role of financial institutions, and agricultural products have been identified. According to the view of the respondents in this study, the input variable had the highest factorial loading, and the variable of credit institutions had the lowest factorial loading in the economic factor.

Regarding the legal indicator, two variables of the laws related to producers as well as the laws related to distributors and exporters have been examined. The results showed that the factorial loading for the laws related to producers had the greatest impact in our structural analysis.

Among the three indicators of education, extension, and research, it was reported that extension indicator had highest factorial loading and research indicators had the lowest factorial loading.

Based on the results of study, two social and technical factors have not had much effect on collaborative networking among cooperatives.

According to the results of the structural equation test illustrated in Table 4, the path coefficients' t-value for policymaking, legal, economic, and educational mechanisms, showed that there was positive relationship between these mechanisms and development of collaborative networking at 0.01 and 0.05 level of significance. The significance of path coefficients for policymaking, and legal mechanisms have been less than 0.01, while for economic, and educational have been less than 0.05. The results show that the strength of structural relationship between policy making mechanisms and development of collaborative networking was higher compared to other mechanisms.

**Table 4:** Estimation of the differences between parameters significance.

Relationship between parameters			Coefficient	Critical ratio	Level of significance
Educational, Extension and research mechanisms∗	→	Development of Collaborative Networking	0/130	2/262	0/026
Social mechanisms	→	Development of Collaborative Networking	0/230	1/194	0/213
Legal mechanisms∗∗	→	Development of Collaborative Networking	0/208	2/839	0/004
Technical mechanisms	→	Development of Collaborative Networking	0/022	0/357	0/712
Policy making mechanisms∗∗	→	Development of Collaborative Networking	0/539	7/881	0/000
Economical mechanisms∗	→	Development of Collaborative Networking	0/499	2/486	0/016

∗∗ 99% level of significance.

∗ 95% level of significance.

It was also observed that values for social and technical mechanisms have been greater than 0.05 and there was no significant relationship between social and technical mechanisms with development of collaborative networking. The results also showed that the social factors and technical mechanisms had weakest structural relationship among all factors.

## 6 Discussion

National governments pursue strategies in which strengthening the cooperative sector is seen as a priority for sustainable economic development. This study examines the mechanisms that can be involved in development of collaborative networking among agricultural cooperatives in the Karaj Province. The results of descriptive analysis of the data show that 68% of the population have bachelor’s degrees. Furthermore, having an average of 18 years of experience in working in cooperatives shows that the members of the population have had enough experience in their profession. The results also show that the target population in this study was dominated by the male population (80%).

This study specifically explored the relationship between independent variables (mechanisms) and dependent variable (collaborative networking). The results indicated a significant relationship between educational, economical, policy making and legal mechanisms and collaborative networking.

One potential mechanism that develop the collaborative networking among cooperatives is economic. That’s because this mechanism has been considered by agricultural policy makers to provide financial services and inputs to the target population. In the context of economic policies, respondents indicated that insurance and emergency policies, subsidies, and guaranteed purchases, could be enhanced establishing collaborative networking among cooperatives. From respondent’s perspective, the input indicator has had the greatest impact on our proposed network model due to its significant role in reducing the end-user price. The result of study by Kumar et al. (2015) about the impact of cooperatives shows that members have benefited economically and financially from their membership in the cooperative in India. Therefore, the establishing of networks among cooperative could eventually increase the benefits and provide better financial services for the members.

In regard to the role of legal mechanisms in development of collaborative networking, it was found out that rules and regulation which create and facilitate the ground to develop collaboration among cooperative needs to be approved and modified by the government to make the process easier and more efficient. The results of study by Woldesenbet (2019) about factors affecting the development of show that cooperatives need to find a stable ground of politico-legal condition as regulatory framework and support policies that are coherent with the co-operative form and favor its development as another factor. Therefore, to support cooperative and act accordingly it must have strong legal ground.

Three variables are used to capture the collaborative networking mechanism namely, network-based behavior, market-oriented behavior, and relationship-based behavior. Considering the role of behaviour, market-oriented behaviour may have more positive ramification in establishing networks among cooperative. This is in line with the study of Shamabadi and Barimnejad (2007) that identifying the factors and variables affecting the marketing of agricultural products highlighted a network of rural cooperatives as a tool for the sale of agricultural products.

The policy making mechanisms was found to play an important role in developing collaborative networking among agricultural cooperatives. This finding is in accordance with the study by Mistris et al. (2020) that shows the role of policy making factor on development of cooperatives in Latvia.

The proposed network has the potential in offering educational services such as training courses required by network members. Educational services have direct and indirect benefits for organizations and their members in the networks and enhance collaboration among them (PHF, 2016). Surprisingly, the respondents believed that the research dimension had a lesser impact on the development of networking than the educational dimension. Perhaps, it may be the structure of cooperative in Iran that research activities, due to its existing capacity constraints, poses a challenge in enhancing collaboration among networks of cooperatives.

The results indicated a non-significant relationship between social mechanisms and development of collaborative networking. Therefore, it can be concluded that social mechanisms have not influenced the development of networking in production cooperatives. Such a result is not in accordance with a study done by Entrnam (2007), who indicated that cultural factors such as the level of social trust, and participation influencing the formation of agricultural production networks.

## 7 Conclusions

There are very limited studies on networking in cooperatives and it is expected that such studies can fill in the gap. In addition, due to the structural and functional differences between cooperatives and other organizations, it seems that the findings of this study can make a significant contribution to providing a ground for building networks between cooperatives.

The results of this study show that economic, educational, policy and legal mechanisms are identified factors that play a role in collaborative networking cooperatives. The result confirmed the hypothesis because it shows that there is a relationship between the variables involved. However, social, and technical mechanisms are not considered as the main factors affecting the collaborative networking among cooperatives, because the results showed a weak positive relationship between the variables.

One of the main outcomes of this study is the promise of cooperative networking to deliver positive consequences for the efficiency and effectiveness of agricultural production cooperatives as well as better service.

Findings of this study by focusing on the role of networking would improve the performance of cooperatives and indirectly helping the government in achieving the economic goals.

The findings of this study will also help policy makers and planners to identify weaknesses and shortcomings to improve the performance of cooperatives and to achieve the goals of the cooperative sector.

The analysis of factors in this study would allow practitioner and policy makers to understand the current pattern of relationships between cooperatives and develop model to improve the collaboration between cooperative through networking.

## 8 Limitation

The practical consequences of this study are limited to agricultural production cooperatives in Karaj province, so the networking in these cooperatives is probably different from other specialized cooperatives as well as agricultural cooperatives in other provinces.

## 9 Further implication of the study

This study has provided results that contributed to cooperation and collaboration among cooperative societies, however there are certain limitations about its applicability in other cooperatives and further research is needed to test its validity.

It is also recommended that studies should be conducted to examine the factors that contribute to the development of informal networks among cooperatives.

The results of this study emphasize that there is need for future studies about internal characteristics of cooperative networks. Furthermore, more research is needed on the critical determinants of the success of these networks.

## Declarations

### Author contribution statement

Mohammad Alimohammad, Seyed Jamal Farajallah Hosseini, Seyed Mehdi Mirdamadi, Sahar Dehyoori: Conceived and designed the experiments; Performed the experiments; Analyzed and interpreted the data; Contributed reagents, materials, analysis tools or data; Wrote the paper.

### Funding statement

This research did not receive any specific grant from funding agencies in the public, commercial, or not-for-profit sectors.

### Data availability statement

Data will be made available on request.

### Declaration of interest’s statement

The authors declare no conflict of interest.

### Additional information

No additional information is available for this paper.

## Appendix A Supplementary data

Questionnaire Questionnaire
