# Supplementary material for: Collaborative networking among agricultural production cooperatives in Iran
Source: Heliyon. 2022 Nov 26;8(11):e11846. doi: 10.1016/j.heliyon.2022.e11846 (PMC9712559; doi:10.1016/j.heliyon.2022.e11846)
Supplement: Questionnaire [file mmc1.doc]

Date:

Questionnaire number:

Dear respondent, greetings:

This questionnaire is related to research titled: "Designing the network model of agricultural production cooperatives in Alborz province". Considering that you are a member of the research statistical community, helping to study and complete the questions of this questionnaire and providing your opinions and views on the questions raised can help the best results of this research. It is necessary to remember that your answers are considered confidential and will be kept by the research committee. Thank you in advance for your sincere cooperation in helping to do this research as well as possible.

Research Committee: Student:

Dr. Seyed Jamal Farajolah Hosseini Mohammad Alimohammad

Dr. Seyed Mehdi Mirdamadi

Dr. Sahar Dehyuri

A- What is the purpose of networking in cooperative companies from your point of view?

| dimensions | number | quaere | Not important | Slightly Important | Fairly Important | Moderately Important | Very Important | Absolutely Important |
| --- | --- | --- | --- | --- | --- | --- | --- | --- |
| Network-oriented behavior | 1 | How effective is the formation of a union? |  |  |  |  |  |  |
|  | 2 | Union activity makes agricultural activity profitable. |  |  |  |  |  |  |
|  | 3 | Working in the union will lead to crisis management. |  |  |  |  |  |  |
|  | 4 | Working in the union leads to improvement in managerial behavior. |  |  |  |  |  |  |
| Market oriented behavior | 5 | Attendance in the union will lead to awareness and timely action on market changes and events. |  |  |  |  |  |  |
|  | 6 | Attendance in the union increases competitiveness in the market. |  |  |  |  |  |  |
|  | 7 | The activity in the union leads to presence in new markets. |  |  |  |  |  |  |
|  | 8 | Working in the union will increase customer attraction. |  |  |  |  |  |  |
| Relationship oriented behavior | 9 | The absence of agricultural activists in the union will cause the union to be ineffective. |  |  |  |  |  |  |
|  | 10 | Attendance in the union strengthens the motivation of joint ventures. |  |  |  |  |  |  |
|  | 11 | Attendance in a union improves the interaction with other business competitors. |  |  |  |  |  |  |
|  | 12 | Attendance in the union will assist you in finding qualified business partners. |  |  |  |  |  |  |

B- What is the role of technical mechanisms in the networking of agricultural cooperatives?

| dimensions | number | quaere | Not important | Slightly Important | Fairly Important | Moderately Important | Very Important | Absolutely Important |
| --- | --- | --- | --- | --- | --- | --- | --- | --- |
| Productivity | 19 | To what extent does the implementation of new production methods contribute to the development of the network? |  |  |  |  |  |  |
|  | 20 | How much does resource consumption management have in network development? |  |  |  |  |  |  |
|  | 21 | Does the reduction of agricultural waste play a role in the development of networks? |  |  |  |  |  |  |
|  | 22 | To what extent does the applying of novel tools and machines contribute to the expansion of the network? |  |  |  |  |  |  |
|  | 23 | To what extent does increasing production efficiency play a role in network development? |  |  |  |  |  |  |
| Production management | 24 | To what extent does the development of infrastructure facilities play a role in networking? |  |  |  |  |  |  |
|  | 25 | How much impact does easy access to technology have on network activity? |  |  |  |  |  |  |
|  | 26 | To what extent does the existence of expert and skilled forces play a role in the development of the network? |  |  |  |  |  |  |
|  | 27 | To what extent are incidents and accidents in the work environment effective in the role of networks? |  |  |  |  |  |  |
|  | 28 | To what extent does having the potential to deal with crises and unexpected incidents play a role in the activities of the network? |  |  |  |  |  |  |
|  | 29 | How much does the use of breeds and varieties contribute to the productivity of networks? |  |  |  |  |  |  |
|  | 30 | To what extent does the compatibility of the quality of the products with the needs of the export market lead to the prosperity of the network? |  |  |  |  |  |  |

C- What is the role of economic mechanisms in the networking of agricultural cooperatives?

| dimensions | number | quaere | Not important | Slightly Important | Fairly Important | Moderately Important | Very Important | Absolutely Important |
| --- | --- | --- | --- | --- | --- | --- | --- | --- |
| inputs | 31 | Price stability can be effective in network activity. |  |  |  |  |  |  |
|  | 32 | The distribution of high-quality inputs is effective in the activity and expansion of networks. |  |  |  |  |  |  |
|  | 33 | Consciousness of the quality of inputs has an impact on the efficiency of networks. |  |  |  |  |  |  |
|  | 34 | Appropriate geographic access to inputs play a role in network activity. |  |  |  |  |  |  |
|  | 35 | Providing government inputs play a role in the development of networks. |  |  |  |  |  |  |
|  | 36 | In times of scarcity of inputs, the role of network activity becomes effective. |  |  |  |  |  |  |
|  | 37 | Distribution of input by networks in the market is more suitable than distribution from networks. |  |  |  |  |  |  |
|  | 38 | The role of networks is decisive in the import of agricultural inputs. |  |  |  |  |  |  |
| agricultural products | 39 | Guaranteed purchase of agricultural products by the government will be effective in network activities. |  |  |  |  |  |  |
|  | 40 | What is the level of satisfaction with the purchase price of agricultural products in networks? |  |  |  |  |  |  |
|  | 41 | A suitable and stable market for buying agricultural products is effective in expanding the network of agricultural cooperatives. |  |  |  |  |  |  |
|  | 42 | The activity of agricultural products transformation industries is effective in the development of networks along with agricultural activities. |  |  |  |  |  |  |
|  | 43 | The presence of intermediaries and dealers in the purchase of agricultural products is effective in the activity of agricultural networks. |  |  |  |  |  |  |
|  | 44 | A suitable export market is effective in the activity of networks. |  |  |  |  |  |  |
|  | 45 | Local markets play a role in the activity of networks. |  |  |  |  |  |  |
|  | 46 | Exhibitions for the supply of agricultural products are effective in the activity of networks. |  |  |  |  |  |  |
|  | 47 | The quality of agricultural products is effective in the production units of the networks. |  |  |  |  |  |  |
| Determining the role of government agencies | 48 | The interaction of government agencies with producers plays a role in the development of networks. |  |  |  |  |  |  |
|  | 49 | Accountability of government agencies to producers play a role in the development of networks. |  |  |  |  |  |  |
|  | 50 | It is more appropriate to monitor government activities by networks. |  |  |  |  |  |  |
|  | 51 | The implementation of infrastructure projects by the government is effective in the development of networks. |  |  |  |  |  |  |
|  | 52 | Fair and appropriate distribution of government credits is effective in the development of networks. |  |  |  |  |  |  |
|  | 53 | The government's financial assistance to the producers will affect the activity of the networks. |  |  |  |  |  |  |
| Determining the role of financial institutions | 54 | The cooperation and interaction of financial and credit institutions affects the activity of networks. |  |  |  |  |  |  |
|  | 55 | Proper distribution of internal resources and banking facilities of financial institutions has a role in the activity of networks. |  |  |  |  |  |  |
|  | 56 | How effective are the activities of guaranteed investment funds in the development of networks? |  |  |  |  |  |  |
|  | 57 | The provision of services by insurance companies to producers will be effective in the development of networks. |  |  |  |  |  |  |

D- What is the role of legal mechanisms in the networking of agricultural cooperatives?

| dimensions | number | quaere | Not important | Slightly Important | Fairly Important | Moderately Important | Very Important | Absolutely Important |
| --- | --- | --- | --- | --- | --- | --- | --- | --- |
| Rules and regulations related to producers | 58 | Passing laws on tax exemptions for manufacturers plays a role in the development of network activities. |  |  |  |  |  |  |
|  | 59 | The approval of laws restricting the import of agricultural products plays a role in the development of networks. |  |  |  |  |  |  |
|  | 60 | The approval of farmers' protection laws will lead to the development of networks. |  |  |  |  |  |  |
|  | 61 | Revision of the existing laws in order to facilitate the activities of farmers will lead to the development of networks. |  |  |  |  |  |  |
|  | 62 | Requiring executive bodies to implement protective laws will support networks. |  |  |  |  |  |  |
|  | 63 | Interaction with international organizations plays a role in the development of networks. |  |  |  |  |  |  |
|  | 64 | Joining the World Trade Organization plays a role in the development of networks. |  |  |  |  |  |  |
|  | 65 | Alignment of existing laws and regulations with international laws plays a role in the development of networks. |  |  |  |  |  |  |
| Rules related to sellers and exporters | 66 | Facilitation of export laws plays a role in the development of networks. |  |  |  |  |  |  |
|  | 67 | Legal support for transformation industries plays a role in the development of networks. |  |  |  |  |  |  |
|  | 68 | Adoption of laws to eliminate middlemen and direct sale of products in the market plays a role in the development of networks. |  |  |  |  |  |  |

E- What is the role of social mechanisms in the networking of agricultural cooperatives?

| dimensions | number | quaere | Not important | Slightly Important | Fairly Important | Moderately Important | Very Important | Absolutely Important |
| --- | --- | --- | --- | --- | --- | --- | --- | --- |
| The role of non-governmental organizations | 69 | The activity of non-governmental organizations and institutions with an agricultural approach plays a role in the development of networks. |  |  |  |  |  |  |
|  | 70 | Government support of networks and organizations is effective in agricultural development. |  |  |  |  |  |  |
|  | 71 | How much does the outsourcing of governance tasks to non-governmental organizations affect the development of networks? |  |  |  |  |  |  |
|  | 72 | Networks have more appropriate executive power than other public institutions in outsourcing governance tasks. |  |  |  |  |  |  |
|  | 73 | Networks have the power of appropriate supervision in the government's sovereign duties. |  |  |  |  |  |  |
| cultural factors | 74 | It is possible to promote the collaborative culture through networks. |  |  |  |  |  |  |
|  | 75 | It is possible to preserve and promote the tribal culture and customs in the field of agriculture through networks. |  |  |  |  |  |  |
|  | 76 | It is possible to promote the culture of using organic products through networks. |  |  |  |  |  |  |
|  | 77 | It is possible to inform consumers about the health of agricultural products through networks. |  |  |  |  |  |  |

F- What is the role of educational, promotional and research mechanisms in the networking of agricultural cooperatives?

| dimensions | number | quaere | Not important | Slightly Important | Fairly Important | Moderately Important | Very Important | Absolutely Important |
| --- | --- | --- | --- | --- | --- | --- | --- | --- |
| educational | 78 | It is possible to assess needs and hold training courses for producers by networks. |  |  |  |  |  |  |
|  | 79 | It is possible to hold theoretical and practical training courses through networks. |  |  |  |  |  |  |
|  | 80 | It is possible to hold virtual training courses through networks. |  |  |  |  |  |  |
|  | 81 | It is possible to provide capable trainers in training through networks. |  |  |  |  |  |  |
| extension | 82 | Networks play a role in transferring new technologies to farmers. |  |  |  |  |  |  |
|  | 83 | Networks play a role in implementing novel promotion plans. |  |  |  |  |  |  |
|  | 84 | Networks are effective in applying books, promotional publications, virtual media, etc. |  |  |  |  |  |  |
|  | 85 | It is possible to exchange information and experiences in Virtual Environment through networks. |  |  |  |  |  |  |
| research | 86 | It is possible to assess the needs assessment of research projects by networks. |  |  |  |  |  |  |
|  | 87 | It is possible to link scientific research centers to the activists of the agricultural sector of the networks. |  |  |  |  |  |  |
|  | 88 | It is possible to implement research projects in the agricultural sector with the assistance of networks. |  |  |  |  |  |  |
|  | 89 | It is possible to implement research projects by international organizations with the companionship of networks. |  |  |  |  |  |  |

G- What is the role of policy-making mechanisms in the networking of agricultural cooperatives?

| dimensions | number | quaere | Not important | Slightly Important | Fairly Important | Moderately Important | Very Important | Absolutely Important |
| --- | --- | --- | --- | --- | --- | --- | --- | --- |
| Economic policy | 90 | Implementing the policy of direct payments (subsidies) to farmers will lead to the development of networks. |  |  |  |  |  |  |
|  | 91 | The implementation of risk management policies such as insurance against unexpected events will support the network's activity. |  |  |  |  |  |  |
|  | 92 | The implementation of the policies of exemptions and legal concessions in the agricultural sector will develop the activities of the networks. |  |  |  |  |  |  |
|  | 93 | The implementation of guaranteed purchase policies of products leads to the development of agricultural networks. |  |  |  |  |  |  |
| Development policy | 94 | Improving transportation mechanisms, road infrastructures, providing civil services to the agricultural business will lead to the development of networks. |  |  |  |  |  |  |
|  | 95 | Improving agricultural security mechanisms will lead to the development of networks. |  |  |  |  |  |  |
|  | 96 | Improving the health and treatment policies of agricultural business environments will lead to the development of networks. |  |  |  |  |  |  |
|  | 97 | Improving the educational and research policies of agricultural business will lead to the development of networks. |  |  |  |  |  |  |

Personal characteristics:

Gender: ِ male ِ female

Age: .............

Educational level: □ Illiterate □ Primary education □ Middle education

□ High school diploma’s degree □ Associates’ degree

□ Bachelor’s degree □ Bachelor’s degree plus (Master’s degree and above)

Production type: ........................

Business activity license: □ Yes □ No

Type of ownership: □ owner □ rental

□ Membership in a cooperative □ Membership in a union

In the end, if you have any additional comments or questions, please write.

...................................................................................................................................................... .

...................................................................................................................................................... .

........................................................................................................................................................

Thank you for your assistance
